# Anti-Inflammatory Characteristics of Local Anesthetics: Inhibition of TNF-α Secretion of Lipopolysaccharide-Stimulated Leucocytes in Human Blood Samples

**DOI:** 10.3390/ijms23063283

**Published:** 2022-03-18

**Authors:** Stefan Weinschenk, Carsten Weiss, Justus Benrath, Volker von Baehr, Thomas Strowitzki, Manuel Feißt

**Affiliations:** 1 Department of Gynecological Endocrinology and Fertility Disorders, Women’s Hospital, University of Heidelberg, D-69120 Heidelberg, Germany; thomas.strowitzki@med.uni-heidelberg.de; 2 Institute of Biological and Chemical Systems, Biological Information Processing, Karlsruhe Institute of Technology (KIT), Campus North, D-76133 Karlsruhe, Germany; carsten.weiss@kit.edu; 3 Pain Clinic, Mannheim University Hospital, Faculty of Heidelberg University, D-68167 Mannheim, Germany; justus.benrath@umm.de; 4 Institute of Medical Diagnostics, Nicolaistraße 22, D-12247 Berlin, Germany; v.vonbaehr@imd-berlin.de; 5 Institute of Medical Biometry (IMBI), Heidelberg University, Im Neuenheimer Feld 130.3, D-69120 Heidelberg, Germany; feisst@imbi.uni-heidelberg.de

**Keywords:** clinical study, cytokines, local anesthetics, LPS, neural therapy, tumor necrosis factor-alpha

## Abstract

Background. Local anesthetics (LAs) have potent anti-inflammatory properties. Inflammatory down-regulation is crucial in diseases with overactive immune reactions, such as acute respiratory distress syndrome (ARDS) and chronic inflammation. We investigated the influence of four LAs, procaine, lidocaine, mepivacaine, and bupivacaine, on the reduction of tumor necrosis factor-alpha (TNF-α) secretion in lipopolysaccharide (LPS)-activated human leucocytes. Methods. Blood samples of 28 individuals were stimulated with LPS. The reduction of TNF-α production by each of the four LAs added (0.5 mg/mL) was measured and correlated with biometric variables. A response was defined as reduction to <85% of initial levels. Results. All four LAs down-regulated the TNF-α secretion in 44–61%: Bupivacaine (44.4%), lidocaine (61.5%), mepivacaine (44.4%), and procaine (50% of the individuals, “responders”). The TNF-α secretion was reduced to 67.4, 68.0, 63.6, and 67.1% of the initial values in responders. The effects in both patients and healthy persons were the same. Interindividual responses to LAs were not correlated with the duration or type of complaints, basal TNF-α serum level, sex, BMI, or age of responders. Conclusions. Four clinically relevant LAs (amid-LA and ester-LA) attenuate the inflammatory response provoked by LPS. They are potential candidates for drug repositioning in treating overactive immune reactions and chronic inflammation.

## 1. Introduction

Local anesthetics (LAs) possess anti-inflammatory properties in in vitro and in vivo [1,2]. LAs can improve treatment results in, for instance, acute pancreatitis [3], distal colitis [4], and recovery from abdominal surgery [5]. In infectious diseases, such as COVID-19, the overactive immune response (“cytokine storm”) becomes more harmful than the infection itself [6]. Thus, tissue protection from damage by immune over-reaction may be crucial in such conditions.

In vitro LAs show strong immuno-modulatory properties. Lidocaine suppresses the secretion of the cytokine TNF-α [7] in human T-cells and in rat microglia cell cultures [8]. In human intestinal cancer cells, lidocaine suppresses the basal and stimulated release of the interleukins IL-8 and 10 independent of voltage-gated sodium channels (VGSC) [9]. Two possible mechanisms mediating this effect have been postulated: Lidocaine inhibits NF-κB-mediated mRNA expression and interferes with the activation of p38 mitogen activated protein kinase (MAPK) [7], two central pathways, which are often induced in parallel and are key regulators of inflammation and a general stress response [10]. Lidocaine down-regulates Toll-like receptor 4 (TLR-4), as was shown in septic rats in in vivo [11] and down-regulates TLR-2 in allergic airway inflammation in mice [12]. Lidocaine and bupivacaine reduce cytokine TNF-α and prostaglandin production in human macrophages and mesenchymal stem cells [13]. In in vivo, lidocaine infusion decreases the elevated TNF-α level in experimental lung surgery in pigs [14]. 

TNF-α is an important factor in inflammation and carcinogenesis. Multiple signaling pathways contribute to the enhanced transcription, translation, and release of TNF-α. Most prominent transcription factors regulating the promotor of TNF-α gene are NF-κB and AP-1, which are downstream targets of the protein kinase family’s MAPK and inhibitor of NF-κB kinase (IKK) [15,16,17]. The inflammatory response, associated with an overproduction of cytokines, correlates with the severity of acute respiratory distress syndrome (ARDS) induced by corona viruses [18]. Elevated TNF-α levels are found in SARS patients with severe lung damage needing intensive care treatment [19]. 

In oncogenesis, TNF-α increases the expression of intracellular adhesion molecules such as ICAM-1. ICAM-1 is a receptor necessary for leukocyte adhesion and tumor invasion. This receptor assists with tumor extravasation via the binding of neutrophils. TNF-α also activates the src protein tyrosine kinase, a regulator of endothelial permeability. Src is a proto-oncogene regulating cell growth involved in the extravasation of tumor cells. Lidocaine and ropivacaine can decrease src activation and ICAM-1 phosphorylation [20]. In various trials, LAs have been shown to possess antineoplastic potential [21]. Prevention of tumor recurrence by the anti-metastatic potential of LAs [20] is currently intensively studied in oncology research [21].

The aim of this study was to expand the research investigating the capability of LAs to regulate important pro-inflammatory pathways. Whereas most previous observations were obtained using transformed (e.g., tumorigenic) cell lines from single donors and animal models, we wanted to provide evidence for the anti-inflammatory activity of LAs in human blood samples as well. We examined various donors, addressing interindividual differences as well as the general conservation of the response. We were able to substantiate the clinical relevance of this concept by the data presented in our investigation. We investigated the widely used LAs lidocaine, procaine, mepivacaine, and bupivacaine for their ability to inhibit the TNF-α production in LPS-stimulated human WBCs, from full blood, especially monocytes. The anti-inflammatory properties of LAs have only been studied in cell lines or animal models so far. We wanted to corroborate such findings in human whole blood samples. We used a well-established assay for analyzing the secretion of TNF-α by WBC, which were stimulated with the TLR4 agonist LPS. In clinical routines, this system is used for detecting individual effects of anti-inflammatory drugs in the respective patient. It is superior to WBC extracts.

We hypothesize that different LAs all directly influence the TNF-α production cycle and are therefore candidates for the suppression of overactive inflammation and tumor recurrence as well. Furthermore, we assume that there may be differences in immunoactivity between different LAs, according to their chemical structure, lipophilicity, stereochemistry, clinical effectiveness (duration of anesthesia), and pharmacokinetics. If LAs can regulate pro-inflammatory pathways, they may be candidates for adjuvant therapy regimes in severely ill SARS patients with an overactive immune response, as well as in patients with chronic (silent) inflammation.

## 2. Results

### 2.1. Characteristics of Individuals

Data from 10 healthy volunteers were available from the dose-finding pretests. The availability of their biometric data was limited due to data protection rules (Table 1). They were 31.6 ± 9.5 years old (range 18.8–49.8 years). The age of the patients was higher than of the healthy individuals. For sex distribution and BMI, there was no difference between patient and healthy individuals.

Eighteen out of 23 consecutive patients who had had therapy-resistant chronic pain or inflammation for ≥6 months were included in this study. The median age of the 18 patients was 61.3 years (SD = 10.4, range 44.7–79.3 years). The median body mass index (BMI) was 24.3 kg/m^2^ (SD = 2.8, range 22.5 to 30.1 kg/m^2^). 

Patients had suffered from chronic pain on average for 10.9 ± 7.8 years (range 6.1–29.1 years). They were treated for chronic pain (N = 13), for chronic inflammation (N = 5), e.g., of the skin, joints, visceral organs (prostate, bladder), and sinusitis. In 14 out of 18 patients, a TNF-α plasma value, a marker for silent inflammation, was available. The average TNF-α value was 6.6 ± 2.3 pg/mL. Twelve of these 14 had normal values, two patients showed a pathologic level > 8.5 pg/mL. The data of all 28 individuals are depicted in Table 1.

### 2.2. Individual Patterns of TNF-α Inhibition

We analyzed possible patterns for each of the LA and for each of the blood samples. To illustrate this, we listed the responses per person and per LA in a “heat map”, see Table 2. A specific pattern of reactivity to LA could not be identified in patients (no. 1–18) nor in healthy individuals (no. 19–28). We also listed moderate inhibition values between 85.5 and 99.9% (light green). Here as well a specific pattern could not be identified. Individuals whose WBC were strongly inhibited by three LAs could nevertheless have no inhibition in the fourth (e.g., patient #18), and vice versa. Due to individual needs (e.g., a suspected allergy to one of the LAs) in some patients and in order to avoid unnecessary economic burden to the patient, not all LAs were tested.

Twenty-three out of 28 persons (82.1%) showed an inhibition of the TNF-α secretion by one or more LA. In the blood of the other five persons there was no inhibition at all (i.e., none of the LA was effective). The blood samples of 21 persons showed an inhibition by 1–3 LAs, and two persons showed an inhibition by all four LAs.

The number of individuals with an inhibition of TNF-α secretion reduced to 85% or more was 12 out of 27 (44.4%) with bupivacaine co-incubation, 16/26 (61.5%) with lidocaine, 12/27 (44.4%) with mepivacaine, and 13/26 (50.0%) with procaine co-incubation (Table 3).

In these responders, the percentage of inhibition by the four LAs was very similar, ranging from 63.6 ± 17.5% to 68.0 ± 13.5%. There was no significant difference between the percentage of inhibition in responders (*p* = 0.63).

### 2.3. Influence of Biometric Factors on TNF-α Inhibition by LAs

We examined the influence of the following covariates on the response rate: Age, sex, and body mass index (BMI), as well as type and duration of the patient’s complaints (Table 4).

Most biometric and anamnestic data did not differ between non-responders and responders for the four LA. We found no significant influence on the response rates based on an individual’s sex, BMI, duration of complaints (correlation = 0.14, *p* = 0.40), type of individual (patient or control), or TNF-α serum levels. However, non-responders were older than responders for procaine (*p* = 0.013), and the number of patients was higher in responders than in the non-responder group for mepivacaine (*p* = 0.049).

## 3. Discussion

### 3.1. LAs Reduce TNF-α Stimulation in White Blood Cells

This pilot study shows that four different local anesthetics, amide-LA and ester-LA, can attenuate the LPS-induced inflammatory response of white blood cells. In more than half of the individual blood specimens, the LAs bupivacaine, lidocaine, mepivacaine, and procaine reduced the LPS-stimulated TNF-α secretion down to 85% or less. Our findings are consistent with cell culture studies, demonstrating reduced LPS-induced TNF-α stimulation by LA co-incubation for lidocaine and bupivacaine [13]. LAs also reduced TNF-α production in LPS-stimulated isolated microglia cells [8]. In this first ex vivo study, we could validate an anti-inflammatory action of LAs in human WBC, thus strengthening the relevance of the preclinical studies performed in cell lines and animal models. 

Because our work is solely focused on TNF-α, its ability to explain the exact mechanism of anti-inflammation is limited. This key cytokine serves as the basic read-out for anti-inflammation in our proof-of-concept study. The most important information to take away from our research is the fact that different LAs have the ability to suppress pro-inflammatory activities in WBC. Further studies investigating different cytokines in human blood derived from healthy donors and patients are warranted to investigate LAs precise site of action. In the following paragraph, we discuss potential pathways addressed by in vitro and animal studies thus far, which could also be relevant in humans.

LAs influence many different receptors other than sodium ion channels [22,23,24,25,26]. All of these effects may contribute to the many different therapeutic effects of LAs [2]. There is increasing evidence that LAs possess important anti-inflammatory properties [1,23,24,25,27,28,29,30]. 

The influence of LAs on the Gαq-protein receptor complex (GPRC) in WBC seems to play an important role in these anti-inflammatory effects [31]. The effect on other T-cell mediated mechanisms is unclear. Lidocaine, an amide-LAs with medium-long analgesic properties inhibits NF-κB-mediated mRNA expression and interferes with the activation of p38 mitogen activated protein kinase (MAPK) [7], two central pathways, being key regulators of inflammation and stress response [10].

The impact of LAs on the NF-κB and the MAPK pathway was demonstrated with lidocaine, an amide-LA, and cocaine, an ester-LA [32]. In our investigation, procaine, as a representative of the ester-LA family, had effects similar to amide-LAs, which was seen by the percentage of individuals with TNF-α inhibition, as well as by the degree of inhibition. Procaine has a very short half-life because it is cleaved immediately by the ubiquitous cholinesterases in almost all tissues. The blood specimens were incubated with LPS and LA for four hours. In our survey, procaine had no lesser effect compared to the three amide-LA. Therefore, procaine (and presumably all other ester-LAs) obviously exerts its anti-inflammatory effect within the first minutes of incubation before it has been degraded by esterases.

### 3.2. Molecular Pathway of the TNF-α Reduction by LA

**Figure 1:**
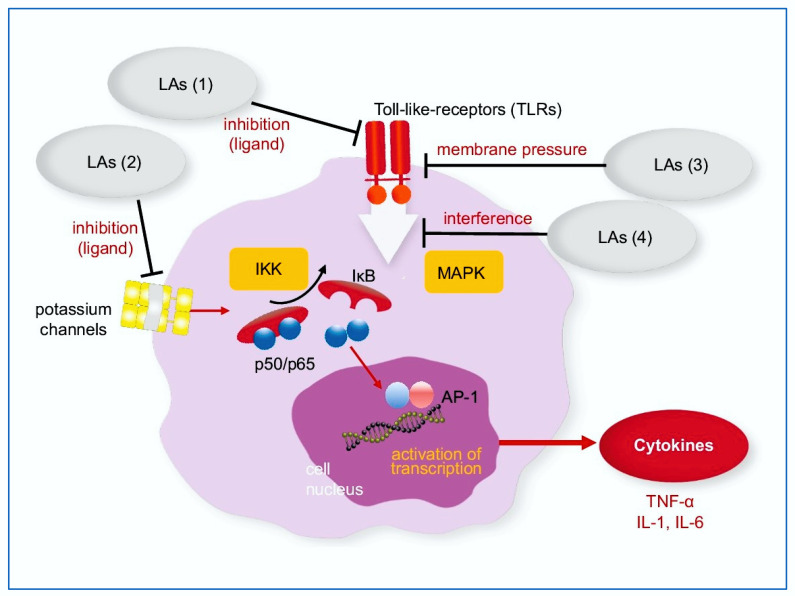
NF-κB pathway: Potential interaction with local anesthetics. LA may inhibit membrane proteins; as TLRs (1) and potassium channels (2), may change the conformation and function of membrane proteins as TLRs by increasing the lateral membrane pressure (3) or may interfere with the NF-κB pathway within the cell (4). IκB: inhibitor of nuclear factor kappa B; IKK: IkBα kinase; IL-1, IL-6: Interleukin-1 and -6, as downstream products of TNF-α, LAs: Local anesthetics; MAPK: mitogen activated protein kinase; TLRs: Toll-like receptors.

Little is known about the exact molecular target of LA inhibiting the production of TNF-α in this complex inflammatory cascade. For lidocaine, Lahat and coworkers showed an inhibitory effect on nuclear translocation of NF-κB in human T-cells [7]. In rat microglia cells, lidocaine not only interfered with nuclear translocation but also with the DNA binding of NF-κB [8]. In addition, the phosphorylation and degradation of the NF-κB-inhibitor, IkBα, was blocked [8]. Therefore, LAs might act on or upstream of the IkBα kinase (IKK) complex. Others proposed an effect on the receptor level important for LPS signaling. However, these findings do not exclude the possibility that LA exert their immune-regulatory effects also via the MAPK pathway. The potential sites of action of LAs on membrane receptors and the NF-κB pathway itself are depicted in Figure 1.

An effect of LAs on toll-like receptors on the cell surface (TLR) has been shown [11]. Activation of the p38 MAPK pathway by LPS was also diminished by co-treatment with LAs [8]. As both signaling cascades, the IKK and MAPK pathways, are downstream of a shared multiprotein complex, which transduces the signal from TLR 4 activation [15]. LA might therefore simultaneously suppress both kinase activities. Lahat et al. also discussed the potential role of potassium channels, which are inhibited by lidocaine and are also important for NF-κB signaling [33,34]. Our assay allows future experiments to distinguish between these two pathways, e.g., by adding MAPK inhibitors, and to unravel the exact mode of suppression of TLR signaling. TLR is a membrane-located receptor. According to the membrane theory of LA [22], LA may influence the TLR even if it does not carry a specific binding site for LA.

About the same number of individuals had an attenuation of TNF-α excretion for all LAs. We would have expected differences, i.e., between patients and healthy individuals, between amide- and ester-LAs, differences correlated with their pKa, their molecular size, or their duration of anesthetic action. Inflammation triggered by GPCRs is reduced by LAs, and the suggested target in this signal transduction pathway is Gαq [31,35]. The LA with the most analgesic potential showed the fewest anti-inflammatory properties and vice versa [27]. These authors describe the relative potencies as follows: Tetracaine > procaine > lidocaine > mepivacaine > bupivacaine > ropivacaine. In our study, all four LAs showed no significant difference in the percentage of responders or in the degree of inhibition. We conclude that interference with the TLR pathway by LAs most probably requires molecular interactions different from GPCR signaling, for example.

### 3.3. Limitations and Strength of the Study

#### 3.3.1. Applicability of the Test System

Measuring TNF-α inhibition has been used by several researchers to evaluate patients’ individual responses to immune-regulatory substances such as mistletoe lectins [36] or herbal extracts like garlic [37] and phyllantus amarus [38]. We routinely use this well-established test system with whole blood-WBCs to evaluate patients’ individual responses to immuno-active drugs. Since 2018, we also apply this test system to LAs. TNF-α is a key cytokine in the inflammation cascade. We did not measure further cytokines in this all-day routine setting for economic reasons. Revealing the precise action site of LA in this anti-inflammatory cascade was not the aim of the study. However, with this data we can provide evidence that LAs also interact with this cascade in WBC from human blood samples of different individuals.

We observed a reduction in more than 50% of the blood samples for all four LAs, which is a far more dramatic effect than we had expected. All four LAs, therefore, are paramount candidates for all kinds of immune-regulative therapies. The test system reveals itself to be an appropriate test also for LA immune function testing.

#### 3.3.2. Why Such Marked Differences between Individuals?

Individual differences of reactions in the TNF-α assay have been shown for many immune-active substances in this test system [36]. In routine testing of other substances, we can often distinguish responders and non-responders. This makes the differences between individuals in LA-induced inhibition unsurprising. Nevertheless, the reasons for these marked individual differences need to be clarified.

#### 3.3.3. Concentration of LAs in the Test System and In Vivo

In pre-tests with dilution series, we found no cell toxicity of the test substances (microscopy with trypan blue staining) in a 1:20 dilution and higher. We therefore can exclude cytotoxic effects of LA on the WBC as a possible reason for the TNF-α inhibition.

A dilution of 1:20 results in a LA concentration of 0.5 mg/mL in the test sample. This is a concentration similar to local tissue concentrations in in vivo. In therapy with LA (TLA, also called neural therapy), the common LA administration is 100 mg LA, resulting in an estimated local tissue concentration of ~5mg in 10–30 mL tissue, which is in the same range (0.5–0.013 mg/mL) as in the test sample, lasting for approximately 3–10 min. Thus, we can assume the amounts and concentrations of LA used in the tests resemble the clinically relevant doses used in daily practice.

#### 3.3.4. Small Observational Study

The advantage of such an observational study is the relevance of the findings for everyday practice. However, because this is a small pilot study with only 28 individuals, we need to be careful with the conclusions on the individual differences of the four LA tested, as well as on the possible covariates of the inhibition of the TNF-α secretion. The difference between the sample size of patients and healthy test persons may be questionable. We chose a 2:1 sample size model because we expected major effects in the patient group. Surprisingly, this was not the case. The high number of responders in both groups to any of the LAs tested confirms immune-regulating properties of LA, independent of the patient’s health status.

Nevertheless, we were able to observe some trends towards a putative influence of covariates. Even if there was no systematic difference between patients and healthy subjects, responders to lidocaine and procaine tended to be younger than non-responders. Conversely, bupivacaine inhibition did not correlate with age, or status, and oddly, mepivacaine showed the opposite effect: Responders were older, and more of them belonged to the patients’ group. Thus, at this time we cannot deduce a correlation of TNF-α inhibition with lifetime in this pilot study. The impact of these covariates on LA’s anti-inflammatory properties may be investigated in further studies based on these findings.

### 3.4. Clinical Implications and Future Investigations

To our knowledge, this inhibition of TNF-α secretion by LAs has not yet been shown in human leucocytes from patients; nor have several different LAs been compared for their immune-regulatory capacity in this important inflammatory pathway.

Our data reveal several questions concerning the immune-regulative characteristics of LA. If all four LAs of different molecular characteristics show TNF-α reduction, is there a correlation between molecule shape, size, chirality, or length of anesthesia, and the impact on the TLR pathway? If not, can the effects be explained by the universal membrane theory of LAs [22]? Can we deduce recommendations to use a specific, reactive LA instead of another one in an individual patient? 

Further studies with specific inhibitors of distinct steps within the MAPK and NF-κB cascades will reveal LA’s site of action (see also Figure 1).

This data also provides a rationale for future clinical studies in chronic inflammation, or in conditions with overactive immune responses, such as SARS. The therapeutic effect of LAs in SARS was not the aim of the study. We therefore did not screen for COVID-19 tests, risk factors, or both in our patients. However, LAs as a co-medication in severe cases of ARDS seem to be a reasonable option [39]. Protection from lung damage by LAs has already been shown in animal models [14,40]. 

### 3.5. TNF-α Testing—A Prognosis of the Therapeutic Potential of LA?

Why might someone be a responder and why might they not? Why do some have a response to a certain LA and why do others not? As soon as we can describe more precisely which target system, and which of the two main pathways, MAPK or NF-κB, will be influenced by LA, we may be able to better explain why some individuals react to one or the other LA and some don’t. However, using this test system, we can already predict the patient’s individual response to a certain LA. Studies correlating the clinical outcome with TNF-α inhibition will further elucidate if recommendations to use a certain LA for therapeutic means are crucial for the therapeutic success. At the moment, it seems reasonable to use the most reactive LA found in an individual’s TNF-α inhibition test for therapy.

## 4. Material and Methods

### 4.1. Dose-Finding Pre-Study and Individuals Tested

We conducted an observational prospective investigation study to assess the inhibition of TNF-α by LAs. Whole blood from 10 healthy volunteers was used for dose-finding studies to determine the outer limits of cytotoxic effects with 1:2, 1:5, 1:10, 1:20, 1:40, 1:80 and 1:160 dilutions of the four LAs. A dilution of 1:20 was found to be non-cytotoxic in all four LAs. From there, this dilution was used in all patient blood specimens. Between April 2018 and March 2019, out of 96 consecutive adult patients with chronic pain of >12 months presenting in an outpatient practice specialized in chronic diseases in Karlsruhe, Germany, 23 patients had therapy-resistant pain: N = 8, vulvodynia; N = 8, chronic pelvic or genital pain; N = 5, musculoskeletal disorders; N = 3, headache/migraine, N = 3 (sum > 100% due to multimorbidity). Five patients suffered from recurrent inflammation. 

Therapy resistance was defined as no further reduction of pain under multimodal therapy for >6 months. Such patients are routinely assessed in our institution to estimate the effects of immune-active substances on cytokine production. We used four LAs frequently used in daily clinical practice: Procaine, an ester-bound LA frequently used in therapy with LA (TLA) [41], and the amid-bound LAs lidocaine, mepivacaine, and bupivacaine. 

Exclusion criteria for usage of patient’s blood specimens in this study were: Acute infectious disorders (e.g., bronchitis, sinusitis, cystitis) (N = 2), current antineoplastic chemotherapy (N = 1) and hyperreactivity to LA (N = 1). LA hyperreactivity was assessed before the beginning of therapy by intracutaneous testing. Furthermore, tests with a minimal basic response of the patient’s blood to LPS stimulation (TNF-α stimulation < 90 percentiles, i.e., 50 pg/mL) were excluded (N = 1).

**Figure 2:**
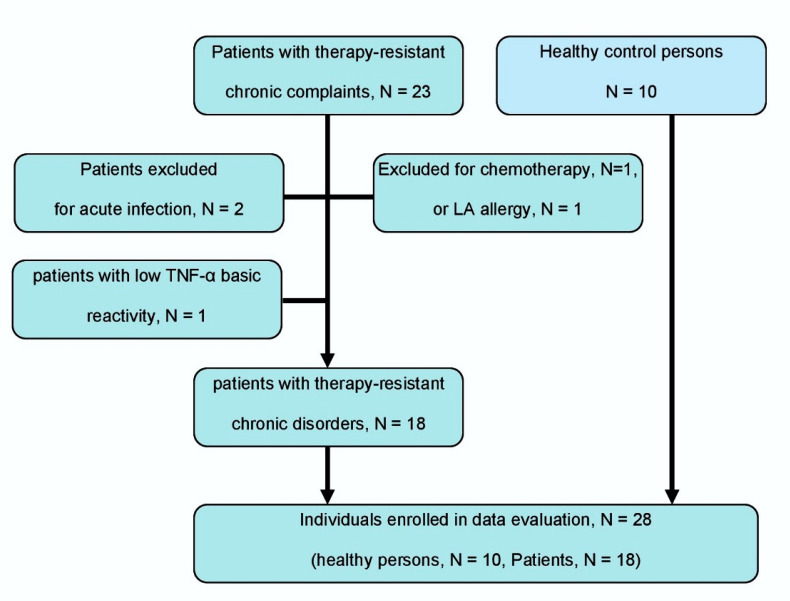
Flow chart of patient and control person selection according to the STARD criteria [42]. Eligible patients are consecutively recruited patients with therapy-resistant chronic pain or inflammation (≥6 months of complaints).

Eighteen out of 23 patients (78.3%) could be enrolled in the study and evaluated. The selection of patients is shown in Figure 2. One or two LAs were not investigated in some patients for practical reasons, due to it being an all-day routine setting. In one of 18 patients, only two of four LAs were tested, and in another 4 patients, only three of four LAs were tested.

### 4.2. Whole Blood Stimulation Test

To test the anti-inflammatory potential of the four LAs, their capacity to inhibit the secretion of the pro-inflammatory cytokine TNF-α was measured in in vitro using a solid-phase chemiluminescence assay. We used an established test system for TNF-α stimulation and inhibition [36,37,38,43,44,45,46]. This system is routinely used for estimating the therapeutic effectiveness of putative immuno-active substances, such as mistletoe lectins [36], indomethacin and acetylic salicylic acid [44], or analgesics [46]. This ex vivo test is superior to WBC extracts compared to peripheral blood mononuclear cell preparations: Whole blood WBC resembles the natural situation in in vivo better than WBC extracts. Most research groups prefer this well-established test system [36,37,38,43,44,45,46].

Induction of TNF-α by LPS was set to 100%. Relative inhibition was considered relevant when the levels were reduced to less than 85%. This cut-off was used by other groups [36,37,38,43,44,45,46] and is a standard cut-off in this kind of test. A virtual increase of stimulation (>100%) in this test is an artifact and counted as “no inhibition”.

From each patient, 50 µL heparinized fresh whole blood was diluted with 500 µL RPMI^®^ cell culture medium (Biochrom, Berlin, Germany) and stimulated in a Falcon^®^ non-pyrogenic 48-well plate (NeoLab Migge, Heidelberg, Germany) with 500 pg/ mL Lipopolysaccharide (LPS O111:B4, Sigma-Aldrich Chemie GmbH, Munich, Germany) and 50 µL saline solution at 37 °C under 5% CO_2_ atmosphere to obtain the value after LPS stimulation.

In this test system, most of the tested substances show cytotoxicity rather than inhibitory effects in concentrations higher than 1:10, see Table 5. To exclude cytotoxic effects by the LAs or by preanalytical handling (e.g., rigorous centrifugation), we performed dose-finding studies with whole blood from 10 healthy volunteers. To detect cell toxicity of the test substances, we microscopically examined the leucocytes after trypan-blue staining [47]. A cell suspension with less than 90% viable cells in this dye test should not be used for cellular function tests. We observed cell toxicity > 10% in LA dilutions of 1:4, 1:8, and 1:10 dilution. The minimal non-toxic dilution in all four LA was 1:20 (LA concentration 0.5 mg/mL), see Table 5. Inhibition effects could be observed in dilutions of 1:20 to 1:80.

In four separate samples, 50 µL of the respective LA in a 1:20 dilution was added instead of saline solution, yielding a final LA concentration of 0.5 mg/mL. After four hours of incubation, TNF-α secretion was measured in the five supernatants (after LPS stimulation without and with one of the four LAs). We used the TNF-α CLIA assay on the IMMULITE^®^ 1000 immunoassay system (Siemens Healthineers, Eschborn, Germany). TNF-α values are given as pg/mL.

### 4.3. Acquisition of Covariates

The patient’s records and the data from healthy individuals (as far as available) were analyzed for the following data: Age, BMI, type of complaints (chronic pain vs. chronic inflammation), duration of complaints at the time of TNF-α test, intake of corticoids, immune suppressors (e.g., MTX) or other chemotherapeutic agents. All patients were non-smokers. All test persons agreed with anonymized data storage and evaluation. 

### 4.4. Statistical Analysis

Statistical analysis was performed using the statistic software R version ≥ 3.5.0 [48]. Successful inhibition was defined as a reduction of the TNF-α level in the supernatant to 85% or less compared to the values obtained after stimulation with TNF-α. Case number estimation had been performed previously on the base of our experience with other substances in routine clinical testing which inhibit TNF-α production. 

Demographic variables were described as frequencies and percentages for categorical variables and as means and standard deviations for continuous variables. Descriptive analysis of the response comprised absolute and relative frequencies of TNF-α inhibition. Results were compared by chi squared tests.

Univariate analysis of possible influence factors for TNF-α inhibition included description by relative frequencies and chi-square tests for categorical variables and mean, standard deviation and Wilcoxon-tests for continuous variables. As this is an exploratory analysis, resulting p-values are to be interpreted in a descriptive sense. *p*-values < 0.05 were defined as significant.

## 5. Conclusions

This study illustrates the novel anti-inflammatory action on the TLR pathway in human blood samples for different LAs, independent of their chemical structure as amide- or ester-LAs. This finding supports a rationale for co-medication studies in chronic inflammation and in conditions with overactive immune response and tissue damage, such as ARDS. Further studies with larger cohorts can be designed on the base of this pilot study.

## Figures and Tables

**Table 1:** Biometric data of patients and healthy individuals.

Covariate	All Individuals	Patients, N = 18	Healthy, N = 10	*p* Value
Number N =	28	18	10	---
Age (years)	49.9 (18.8–79.3)	62.5 (42.4–79.3)	34.8 (18.8–49.8)	<0.001
Sex = female	20 (71.4%)	12 (66.7%)	8 (80%)	0.454
Body mass index	24.2 (19.4–30.1)	24.2 (19.4–30.1)	24.1 (20.4–25.4)	0.632
Chronic pain disorder	13	13	0	---
Recurrent inflammation	8	8	0	---
Serum TNF-α > 8.5 pg/mL	2	2	n. a.	---

**Table 2:** Inhibition of TNF-α release in all patients. The influence of the four LAs is depicted as a “heat map”. Numbers show the TNF-α secretion after adding the respective LA as a percentage of the LPS-induced TNF-α secretion without LA. Inhibition down to 85% or less of the LPS induced secretion (i.e., marked inhibition) is shown in dark green, inhibition down to 85.5–99.9% (moderate inhibition) in light green, and values of 100% and more (no inhibition) in red. White fields: not tested. A virtual stimulation increase (>100%) in this test is an artifact and counted as “no inhibition”.

Person	Patient/Control	Bupivacaine (%)	Lidocaine (%)	Mepivacaine (%)	Procaine (%)
1	Patient	75	61	66	82
2	Patient	126	108	87	94
3	Patient	121	136	106	93
4	Patient	63	65	100	101
5	Patient	61	67	65	77
6	Patient	90	90	109	117
7	Patient		81		96
8	Patient	85	48	43	49
9	Patient	72	83	74	103
10	Patient	93	96	82	104
11	Patient	144	92	87	82
12	Patient	73	105	54	
13	Patient	121	103	97	109
14	Patient	49	85	80	
15	Patient	79		81	90
16	Patient	96		88	137
17	Patient	95	59	82	80
18	Patient	134	20	52	68
19	Test person	92	51	98	54
20	Test person	200	69	166	74
21	Test person	106	77	100	60
22	Test person	57	155	60	81
23	Test person	92	98	117	38
24	Test person	54	43	96	61
25	Test person	77	76	102	122
26	Test person	69	83	56	88
27	Test person	86	50	85	133
28	Test person	96	110	93	68

**Table 3:** Number of responders with a ≥85% reduction of TNF-α-production of the WBC after addition of bupivacaine, lidocaine, mepivacaine, or procaine to the LPS-stimulated whole blood sample.

	Bupivacaine	Lidocaine	Mepivacaine	Procaine	*p*-Value
All persons	44.4%	61.5%	44.4%	50.0%	0.562
Patients	47.1%	56.3%	58.8%	37.5%	0.607
Healthy individuals	40.0%	70.0%	20.0%	70.0%	0.066
p-value (between groups)	0.722	0.483	0.049	0.107	

**Table 4:** Covariates of TNF-α inhibition. Possible influence factors are marked with an asterisk *.

Variable	Bupivacaine			Lidocaine			Mepivacaine			Procaine		
	NR	Resp	*p* Value	NR	Resp	*p* Value	NR	Resp	*p* Value	NR	Resp	*p* Value
Age (mean)	48.49	47.14	0.981	52.10	43.94	0.384	43.17	53.79	0.113	59.22	41.97	0.013 *
Sex (% female)	80.00	66.67	0.432	70.00	68.75	0.946	66.67	83.33	0.326	69.23	69.23	1.000
BMI (mean)	23.97	23.86	0.884	24.62	23.86	0.493	24.00	23.82	0.393	24.59	23.58	0.342
Patients vs. Controls (% Pat)	60.00	66.67	0.722	70.00	56.25	0.483	46.67	83.33	0.049 *	76.92	46.15	0.107
Type of Complaints (% with inflammation)	12.50	50.00	0.124	33.33	42.86	0.725	16.67	37.5	0.393	25.00	20.00	0.835
Duration of Complaints (mean)	9.04	14.15	0.401	9.10	12.69	0.617	10.62	11.69	0.948	11.54	11.32	0.826
Serum TNF-α (mean)	5.63	7.83	0.155	6.12	6.30	1.000	6.20	6.94	1.000	7.086	6.18	0.607

BMI: Body mass index; NR: Non-responder; Pat.: Patients; Resp: Responder.

**Table 5:** Dilution series to determine the non-cytotoxic dilution of the tested four LAs. * = cytotoxic effects found in trypan-blue staining microscopy. No cytotoxic effects, but inhibition was seen in dilutions of 1:20 and higher in all of the LAs tested.

Bupivacaine	Basal	1:2	1:5	1:10	1:20	1:40	1:80	1:160
P1	184	23.5 *	94.3	--	99.4	--	--	177
P2	402	394	294	--	308	--	--	506
P3	1844	102 *	99.4 *	--	1276	--	--	1642
P4	1216	19.3 *	943	--	1044	--	--	1038
P5	833	66.2 *	41.3 *	--	622	--	--	772
P6	1034	955	983	--	1018	--	--	965
**Lidocaine**	Basal	1:2	1:5	1:10	1:20	1:40	1:80	1:160
P1	263	--	--	183 *	244	387	304	299
P2	293	--	--	169 *	293	244	214	292
P3	1183	--	--	926	1296	990	1001	1077
P4	943	--	--	656	701	632	692	891
P5	891	--	--	93 *	782	621	606	837
P6	772	--	--	523	479	417	407	622
P7	875	--	6,7 *	662	669	--	933	--
P8	1844	--	1021	1737	1528	--	1793	--
P9	1216	--	481	617	613	--	1027	--
P10	832	--	11.8 *	902	915	--	892	--
**Mepivacaine**	Basal	1:2	1:5	1:10	1:20	1:40	1:80	1:160
P1	191	--	--	79.1 *	102	155	142	177
P2	281	--	--	170	214	231	219	226
P3	286	--	--	256	214	291	367	341
P4	723	--	--	154 *	326	344	329	487
P5	1735	--	--	943	952	893	1236	1105
P6	1022	--	--	734	693	702	811	1001
P7	1230	5,7 *	6.1 *	--	742	--	1312	--
P8	522	94,2 *	602	--	612	--	570	--
P9	184	18,1 *	194	--	177	--	191	--
P10	402	30,5 *	402	--	409	--	422	--
**Procaine**	Basal	1:2	1:5	1:10	1:20	1:40	1:80	1:160
P1	385			325	317	410	364	388
P2	282			187 *	237	292	299	287
P3	150			84 *	176	170	173	159
P4	832			122 *	456	495	422	820
P5	1253			788 *	622	612	988	1014
P6	928			688	692	701	821	1011
P7	801	--	73.2 *	--	433	--	--	501
P8	473	--	94.4 *	--	348	--	--	384
P9	875	--	422	--	522	--	--	839
P10	1230	--	91.2 *	--	992	--	--	1021

P: person; Basal: TNF-α value on stimulation with LPC without addition of LA. *: cytotoxic effects seen in trypan blue staining microscopy. --: Not tested in this dilution. For bupivacaine, only 6 individuals could be tested.

## Data Availability

All data supporting reported results can be found within the text of the manuscript.

## Abbreviations

ARDS	acute respiratory distress syndrome
BMI	body mass index
IKK	inhibitor of κB kinase
GPCR	G-protein coupled receptor
LAs	Local Anesthetics
LPS	lipopolysaccharide
MAPK	mitogen activated protein kinase
NAS	nominal analogue scale
SARS	severe acute respiratory syndrome
TLA	therapy with Local Anesthetics, Therapeutic Local Anesthesia
TLR	toll-like receptor
TLR4	toll-like receptors 4
TNF-α	tumor necrosis factor-alpha
VGSC	voltage-gated sodium channels
